# Measurement of Lag-Screw Anteversion With an iPhone During Trochanteric Fracture Surgery

**DOI:** 10.7759/cureus.33110

**Published:** 2022-12-29

**Authors:** Yo Kinami, Joe Hasei, Kazuo Fujiwara

**Affiliations:** 1 Department of Orthopaedic Surgery, Okayama City Hospital, Okayama, JPN; 2 Department of Medical Information and Assistive Technology Development, Okayama University Graduate School of Medicine, Dentistry and Pharmaceutical Sciences, Okayama, JPN; 3 Department of Orthopedic Surgery, Okayama City Hospital, Okayama, JPN

**Keywords:** iphone, smartphone, femoral rotation, lag screw, anteversion, table-to-plane, malrotation, trochanteric fracture

## Abstract

Introduction

A useful way to easily evaluate femoral rotation during surgery for trochanteric fractures is not known. Hence, this pilot study aimed to develop an intraoperative indicator to evaluate anteversion in femoral trochanteric fractures.

Material and methods

Prospectively, from June 2021 to January 2022, all patients with femoral trochanteric fractures (Orthopaedic Trauma Association classification: 31A1-3) treated using a cephalo-medullary nail with a lag-screw neck-shaft angle of 125° were included in this study. During surgery, lag-screw anteversion (LS-AV) was measured using the goniometer application in an iPhone with the fractured femur table-top-plane level with the traction table floor. Accuracy was analyzed by comparing axial-projected lag-screw anteversion (AxP-LS-AV) and three-dimensional computed tomography lag-screw anteversion (3DCT-LS-AV) measurements after surgery.

Results

Fifty patients (14 males and 36 females) were included in the study. The mean age was 87 (range; 69-98) years; the Orthopaedic Trauma Association classifications were A1 (28 patients), A2 (18 patients), and A3 (4 patients). The mean LS-AV was 10.7° ± 6.9°, the mean AxP-LS-AV was 12.8° ± 8.3°, and the mean 3DCT-LS-AV was 13.1° ± 8.6°. The median difference between AxP-LS-AV and 3DCT-LS-AV was 3.0° (range: 0°-12°), and 40 (80%) patients had differences of ≤5° (Bland-Altman plot: inside of limit of agreement = 86%, paired *t*-test *p* = 0.7, Pearson correlation coefficient *r* = 0.817, *p *<0.001).

Conclusion

Femur malrotation is defined as a deformity of >15° relative to the normal contralateral limb. Intraoperative LS-AV iPhone measurement on table-top-plane standard had sufficient accuracy as an indicator of anteversion in femoral trochanteric fractures.

## Introduction

Malrotation of femur fractures has also been shown to result in clinically significant functional impairments including decreased functional outcome scores, decreased tolerance for stair climbing, and decreased ambulation endurance [1-4]. The acceptable range of bilateral differences in the femoral rotation is ≤15° [1,2,4-7]. The malrotation>15° has been found to be 17%-28% in femoral shaft fractures [1,2,5-7] and 25.7%-40% in femoral trochanteric fractures [8-11]. The indicators of rotation are “cortical step sign,” “lesser trochanter shape sign,” and” hip lateral view difference,” etc. when treating shaft fractures [12-15].

However, there is no indicator of rotation when treating trochanteric fractures during surgery. The incidence of malrotation of trochanteric fractures is not different between young surgeons and experienced surgeons [11]. The incidence of internal malrotation was higher than the incidence of external malrotation in trochanteric fractures [11]. While treating trochanteric fractures using a cephalo-medullary nail, over-reduction of the proximal fragment of the distal cortex is recommended to prevent excessive sliding which causes fixation failure [16,17]. Internal rotation position for the over-reduction during surgery may cause internal malrotation in trochanteric fractures, while the malrotation cannot be perceived because of no indicator of rotation.

The femoral anteversion cannot be measured directly during surgery using radiography or fluoroscopy. However, we hypothesized that measurement of lag-screw anteversion using a smartphone has potential as an indicator of femoral anteversion. Although some previous studies have described the method for evaluating femoral anteversion during surgery of shaft fractures using smartphones [18-20], no study has yet evaluated femoral anteversion during surgery of trochanteric fractures using a smartphone.

This pilot study aimed to develop an intraoperative indicator for the evaluation of femoral anteversion in trochanteric fractures. We only validated the accuracy of the smartphone technique in this study.

## Materials and methods

This prospective study was conducted at a single-level-2 trauma center, and according to the Declaration of Helsinki. The Research Ethics Committee of Okayama City Hospital issued approval (Approval number 3-146) for the study. Informed consent was obtained in the form of an opt-out on the website.

From June 2021 to January 2022, all patients with femoral trochanteric fractures (Orthopaedic Trauma Association classification: 31A1-3 [21]) treated using a cephalo-medullary nail with a lag-screw neck-shaft angle of 125° were included in this study. IPT-EF (HOMS, Nagano, Japan) nails were used for all patients. Six orthopedic surgeons measured lag-screw anteversion (LS-AV) in their surgery.

The protocol was formulated using three steps: “Decision of reference position/image before surgery,” “Intraoperative measurement of lag-screw anteversion,” and “Evaluation after surgery.”

Decision of reference position/image before surgery

**Figure 1:**
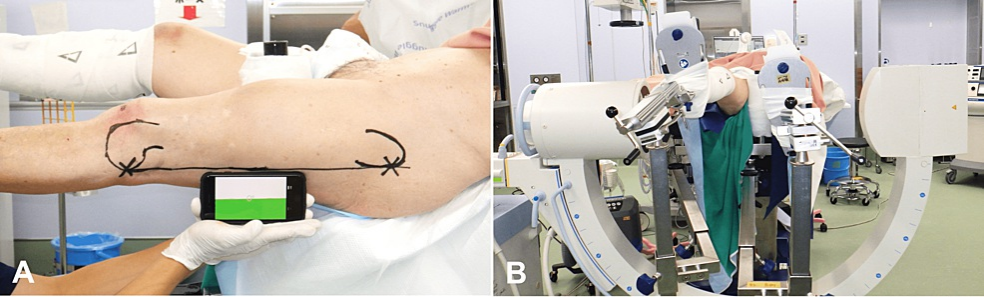
Preparation for setting reference position In the scissors position on the traction table, the normal femur was positioned above the fractured femur to prevent the overlap of the lateral view of both knees [A/B]. The fractured femur was positioned with the patella facing upward and pulled slightly by traction. The posterior edge of the greater trochanter and lateral condyle of the fractured femur were identified by palpation. These two posterior edges were connected with a line using a permanent marker. The fractured femur was positioned at this line, level with the floor, while checking the iPhone’s goniometer application [A]. Next, the image intensifier was set to the horizontal lateral view [B].

The limbs were placed in a scissors position on the traction table (ALPHAMAXX, MAQUET, Rastatt, Baden-Wurttemberg, Germany). The normal femur was positioned above the fractured femur to prevent the overlap of the lateral view of both knees (Figure 1). The fractured femur was positioned with the patella facing upward and pulled slightly by traction. The posterior edge of the greater trochanter and lateral condyle of the fractured femur were identified by palpation. These posterior edges were connected with a line using a permanent marker. The fractured femur was positioned at this line, level with the floor, while checking the goniometer application of iPhone (Apple, Cupertino, CA, USA) (Figure 1A). Next, the image intensifier was set to the horizontal lateral view (Figure 1B)

**Figure 2:**
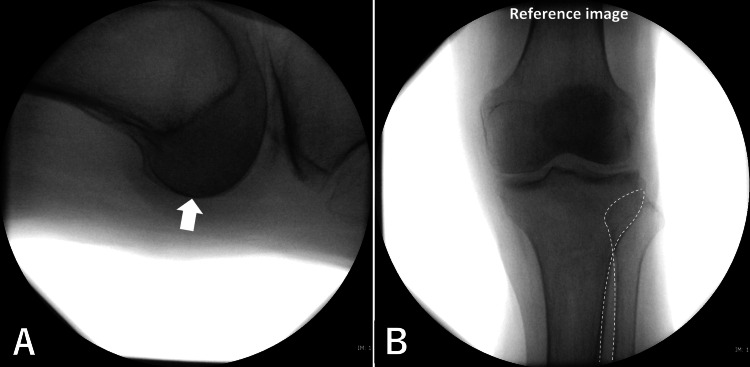
Reference position/image While setting the image intensifier to the horizontal lateral view, the rotation of the fractured femur was positioned so that posterior condylar outlines are positioned to achieve complete overlap and centered (white arrow) on the fluoroscopic monitor [A]. Then, the reference position is set up. Finally, in the same position, the knee anterior–posterior view image is saved for the reference image, and the tibiofibular joint lines (white dotted lines) are recommended to be used as an indicator for rotation [B].

While setting the image intensifier to the horizontal lateral view, the rotation of the fractured femur was positioned so that the posterior condylar outlines overlapped completely and were centered on the fluoroscopic monitor (SIREMOBIL Compact L, SIEMENS, Munich, Bavaria, Germany) (Figure 2A). At this time, the table-top-plane of the fractured femur was level with the floor. The table-top-plane is defined by the posterior edge of the greater trochanter and both condyles. This position was defined as the “reference position” in this study. This lateral knee view was saved for verification. The fluoroscopic knee anterior-posterior view was saved as the “reference image” (Figure 2B) in this reference position. After saving the reference image, the scissors position was changed to a surgical position (contralateral frog-leg position).

Intraoperative measurement of lag-screw anteversion

**Figure 3:**
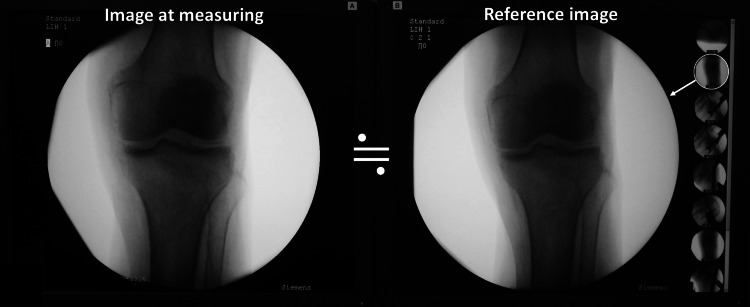
Reappearance of reference position/image The fractured femur was positioned to the table-top plane, level with the floor again. The fractured femur was rotated to afford the anterior-posterior view of the fluoroscopic knee on the monitor similar to the “reference image” while comparing on twin monitors.

After nail fixation on the surgical position during surgery, the fractured femur was positioned so that the marker line connecting the lateral posterior edges was level with the floor again. Subsequently, the rotation of the fractured femur was positioned so that the anterior-posterior view of the fluoroscopic knee on the monitor was similar to the “reference image” while comparing on twin monitors (Figure 3). Furthermore, the “reference position” was set up again. The anterior-posterior view of this knee was saved for verification.

**Figure 4:**
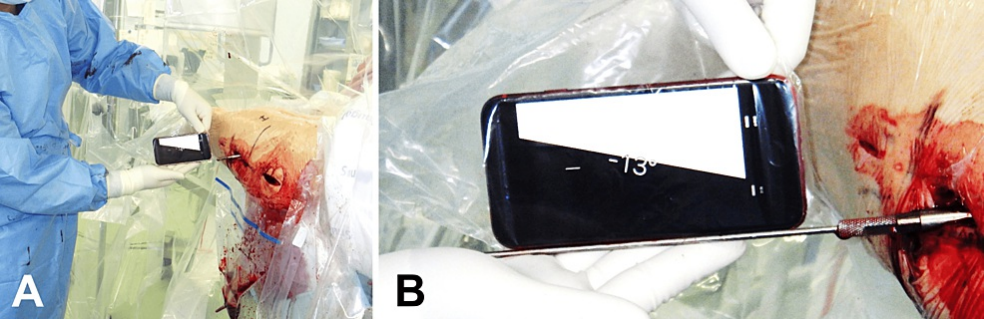
Measurement of LS-AV An iPhone’s goniometer application was opened. the iPhone was packaged in a sterilized transparent bag. The iPhone’s goniometer application was zeroed on the instrument table. The lag-screw anteversion (LS-AV) was measured along the supported guide pin [A]. The level line must cross the figure (example: 13°) [B].

The goniometer application for iPhone with an integrated gyroscope was used to measure LS-AV. An iPhone packaged in a sterilized bag was used, and the iPhone’s goniometer application was zeroed. LS-AV was measured by the iPhone with support provided by a guide pin (Figure 4) and recorded in the surgical record.

Evaluation after surgery

**Figure 5:**
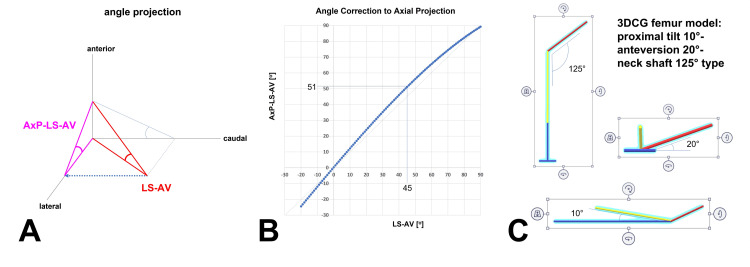
Converting LS-AV to AxP-LS-AV Angle projection [A]: Relationship between the lag-screw anteversion (LS-AV) angle and axial-projected lag-screw anteversion (AxP-LS-AV) angle. Angle-correction graph [B]: Angle-correction graph converting LS-AV to AxP-LS-AV. This graph was specific for implants with a neck-shaft angle of 125°. For example, LS-AV 45° was corrected to AxP-LS-AV 51°. 3DCG femur model [C]: The plot points of the graph were determined from simulation analysis by three-dimensional computer graphics (3DCG). Paint3D software installed in Windows 10 was used to create the 3DCG femur model with a neck-shaft angle of 125°.

LS-AV is corrected to axial-projected lag screw anteversion (AxP-LS-AV) (Figure 5A). The correction is calculated from a graph (Figure 5B). The quick chart for correction is used for rough calculation (Table 1). Plot points of the graph were determined from simulation analysis by three-dimensional computer graphics (3DCG) (Figure 5C).

**Table 1:** Quick chart for correction axial projection angle for normal anteversion range. 5° interval, neck-shaft angle 125°model. (unit = °). LS-AV: lag screw anteversion.  AxP-LS-AV: axial projected lag screw anteversion

LS-AV	-10	-5	0	5	10	15	20	25	30	35	40
AxP-LS-AV	-12	-6	0	6	12	18	24	30	35	41	46
difference	2	1	0	1	2	3	4	5	5	6	6

**Figure 6:**
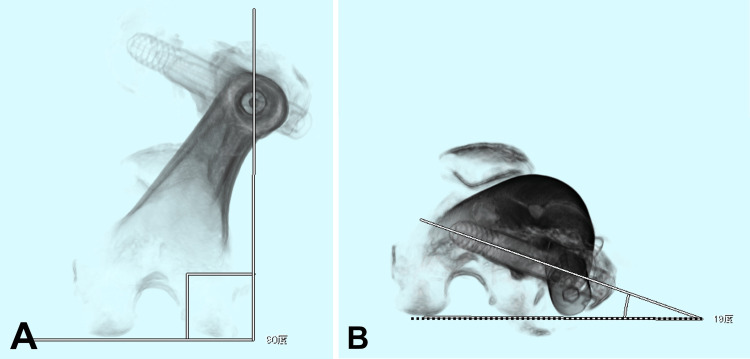
Measurement of 3DCT-LS-AV Three-dimensional computed tomography (3DCT) subjects reconstructed by Synapse Vincent Volume Analyzer: In the cranial view, the distal portion of the nail is initially positioned to zero for abduction/adduction [A]. Next, by vertical rotation around the posterior condyle axis, the posterior edge of the nail end and both posterior condyles are set on a straight line (NE-BPC line), and the three-dimensional computed tomography lag-screw anteversion (3DCT-LS-AV) is defined as the angle between the lag-screw axis and NE-BPC line (dotted line) [B]. The NE-BPC line approximates the table-top-plane line.

Three-dimensional computed tomography (CT) lag screw anteversion (3DCT-LS-AV) is measured after surgery. The 3DCT-LS-AV is defined as the angle between the lag-screw axis and posterior edge of the nail end and both posterior condyles (NE-BPC) lines (Figure 6). SOMATOM Force (SIEMENS, Munich, Bavaria, Germany) was used for computed tomography imaging. The Synapse Vincent Volume Analyzer (FUJIFILM, Tokyo, Japan) was used to reconstruct each three-dimensional subject, and the Synapse Radiology Information System (FUJIFILM, Tokyo, Japan) was used to calculate each 3DCT-LS-AV using digital measurements.

Statistical analysis

Descriptive statistics were described as follows: mean and standard deviation of angle measurements, and median and range of difference between AxP-LS-AV and 3DCT-LS-AV. Bland-Altman plot, paired t-test, and Pearson correlation coefficient were used for the evaluation of accuracy. Statistical analysis was performed using EZR analysis software v1.5 (The R Foundation for Statistical Computing) [22] and Modified R Commander v4.0.2 (for Windows; https://personal.hs.hirosaki-u.ac.jp/pteiki/research/stat/R/).

## Results

**Figure 7:**
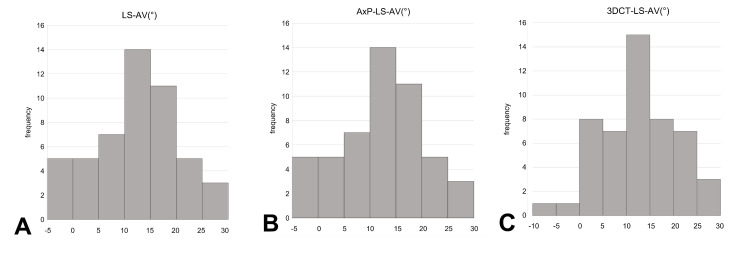
Histogram of angle measurements Histogram of lag-screw anteversion (LS-AV), the mean LS-AV is 10.7° ± 6.9°[A]. Histogram of axial-projected lag-screw anteversion (AxP-LS-AV), the mean AxP-LS-AV is 12.8° ± 8.3° [B]. Histogram of three-dimensional computed tomography lag-screw anteversion (3DCT-LS-AV), the mean 3DCT-LS-AV is 13.1° ± 8.6° [C].

Fifty patients (14 males and 36 females) were included in the study. The mean age was 87 years (range: 69-98 years). The Orthopaedic Trauma Association classification was A1: 28 patients, A2: 18 patients, and A3: 4 patients. The mean LS-AV was 10.7° ± 6.9°. The mean AxP-LS-AV was 12.8° ± 8.3°. The mean 3DCT-LS-AV was 13.1° ± 8.6° (Figure 7).

**Figure 8:**
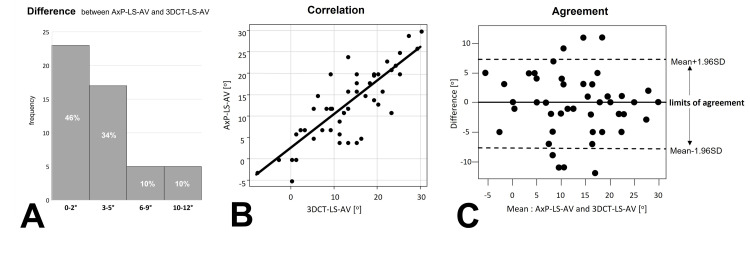
Difference and correlation and agreement, between AxP-LS-AV and 3DCT-LS-AV [A] Histogram of the difference between the axial-projected lag-screw anteversion (AxP-LS-AV) and three-dimensional computed tomography lag-screw anteversion (3DCT-LS-AV): The median difference between AxP-LS-AV and 3DCT-LS-AV is 3.0° (range: 0°–12°), and 40 (80%) patients had a difference of ≤5°. [B] Correlation shows Pearson correlation coefficient r = 0.817, p < 0.001. [C] Agreement of Bland–Altman plot (SD: standard deviation): 43 (86%) patients were within the limit of agreement. 7(14%) patients who were outside the limit of agreement had no similarity between anterior–posterior view of the knee and reference image when measuring lag screw anteversion.

The median difference between AP-LS-AV and 3DCT-LS-AV was 3.0° (range: 0°-12°), and 40 (80%) patients had differences of ≤5°. There were no significant differences between the AxP-LS-AV and 3DCT-LS-AV results (p = 0.7, paired t-test), which were strongly correlated (Pearson correlation coefficient r = 0.817, p < 0.001). Bland-Altman plot revealed that 43 (86%) patients were within the limit of agreement. (Figure 8)

## Discussion

Assessment of result

The lack of a significant difference (paired t-test) between AxP-LS-AV and 3DCT-LS-AV and the strong correlation of Pearson's correlation coefficient (r = 0.817) suggests that AxP-LS-AV can be used intraoperatively to estimate measurements similar to those of 3DCT-LS-AV.

The median difference between these two angle measurements was 3.0°, with a difference of ≤5° in 40 (80%) patients. These results suggest that this iPhone method on table-top-plane standard has sufficient potential as an indicator to derive a bilateral difference of ≤15° in the femoral anteversion angle intraoperatively, based on the preoperatively measured normal femoral anteversion.

The Bland-Altman plot was used to evaluate the agreement between the two angle measurements, which was within the acceptable range in 43 (86%) patients. Postoperative confirmation showed that in 7 patients (14%) who fell outside the acceptable range, the anterior-posterior view image of the knee and the reference image were inconsistent during LS-AV measurements. This result suggests that an agreement between both the images is very important for this method.

Assessment of protocol

Decision of Reference Position/Image Before Surgery

Femur positioning is very important during measurements. Anteversion of the femur is affected by the coronal neck-shaft angle and axial neck axis, which causes variable measurements caused by limb position and poor repeatability [23].

The reference position was defined as the table-top-plane position in this study. The table-top-plane is defined as the plane formed by 3 points: “ the posterior edge of the great trochanter and both posterior condyles,” which is same as the femoral retrocondylar coordinate system taken from the guidelines for three-dimensional evaluation of total hip arthroplasty from Computer Assisted Orthopedic Surgery Japan [23]. This is the definition of the standard measurement of femoral anteversion in Japan and was applied to fracture treatment in this study.

For the reference position, posterior condylar outlines should be positioned with complete overlap and centered on the lateral view of the fluoroscopic monitor (Fig. 2A). Because the fluoroscopic beam has diffusibility, if the posterior condyles are not centered on the monitor, their axes will not be level with the floor.

The anterior-posterior view of the knee was defined as the reference image in this study (Fig. 2B). The knee lateral view is more accurate than the knee anterior-posterior view for a reference image. However, the knee lateral view cannot be observed by fluoroscopy with the patient in the surgical position (contralateral frog-leg position). Changing to the scissors position during surgery consumes time and labor while maintaining a sterile environment.

Intraoperative Measurement of Lag-Screw Anteversion

If the fractured limb was placed in the raised position during surgery, the “table head up” could achieve the reference position easily. Abduction/adduction did not affect the measurement of LS-AV during surgery.

The anterior-posterior view of fluoroscopic knee on the monitor must be as accurately similar to the “reference image” as possible (Fig. 3). The proximal tibiofibular joint outlines were recommended as indicators to control limb rotation. (Fig. 2B). On verification after surgery, the anterior-posterior view of the knee was not similar to the reference image in cases that were outside the limit of agreement, as analyzed using Bland-Altman plot (Fig. 8C).

The iPhone 7 and 8 models were used in this study for measuring LS-AV during surgery. Anteversion of the femur is defined as the angle between the posterior condylar line and neck axis on CT. However, measurement of the neck axis is impossible by X-ray radiography or fluoroscopy during surgery. The authors [18-20] recommended the use of a smartphone to measure femoral anteversion. The procedure in these studies was used for femoral shaft fractures without neck fractures, but the use of a smartphone has been a very useful improvement. The iPhone has an integrated gyroscope and a high-quality goniometer application and is used worldwide. Therefore, angle measurements made by an iPhone are accurate and repeatable.

Evaluation After Surgery

Direct comparison of LS-AV and 3DCT-LS-AV is inadequate, because the 3DCT-LS-AV angle is the axial-projected LS-AV angle (Fig. 5A). Therefore, an angle-correction graph is necessary for axial projection. The correction was determined from a graph generated from simulation analysis using a 3DCG model (Fig. 5B). The Paint3D application in Windows 10 was used to create the neck-shaft angle 125° 3DCG femur model (Fig. 5C). The plot points of the graph were generalized from the value obtained by moving the neck-shaft angle 125° 3DCG model. An approximate formula was created, and the accuracy of the plot points was confirmed.

However, the graph (Fig. 5B) could not be easily used to convert each angle. The quick chart was useful for rough calculations (Table 1). The relation between each angle demonstrated one sequence on interval 5°. This knowledge is useful. To convert AxP-LS-AV, LS-AV 0°-4° are added 0°, LS-AV 5°-9° are added 1°, LS-AV 10°-14° are added 2°, LS-AV 15°-19° are added 3°, LS-AV 20°-24° are added 4°, LS-AV 25°-34° are added 5°, LS-AV 35°-44° are added 6°, based on the quick chart. We used these rough calculations in this study.

Evaluation of anteversion of the femur is more accurate by 3DCT than two dimensional computed tomography (2DCT) [24,25]. Because femur anteversion is defined with the neck axis and posterior condyles, limb positions, such as flection/extension and abduction/adduction, affect 2DCT measurements. The limb position of all data can be converted to the same position on 3DCT.

The NE-BPC line was used by substituting the table-top plane line for measuring anteversion angle on 3DCT. In the cranial view, the distal portion of the nail was initially positioned to zero for abduction /adduction (Fig. 6A). Next, by vertical rotation around the posterior condyle axis, NE-BPC was set on a straight line (Fig. 6B). The NE-BPC line approximated the table-top-plane line. Trochanteric fractures have frequently displaced greater trochanteric fragments. Therefore, the table-top-plane line is not a standard and is not repeatable on 3DCT for trochanteric fractures.

Additional suggestions

We performed 3DCG simulation analysis on two other neck-shaft angle models. LS-AV 45° was corrected to AxP-LS-AV 52° in the neck-shaft angle 130° model and to AxP-LS-AV 53° in the neck-shaft angle 135° model, which gave the same shape graph in each angle model. Therefore, this method may be applied to the neck-shaft angle 130° and neck-shaft angle 135° by calibration using the angle-correction graph of the neck-shaft angle 125° model (Figure 5B).

This iPhone method increased the operative time by about 5-10 min and the fluoroscopic time by a few minutes, for preparation before surgery and measurement during surgery, respectively. However, we think that the benefit of the indicator of anteversion is superior to the drawback of time extension, and that the use of CT after surgery may be avoided.

**Figure 9:**
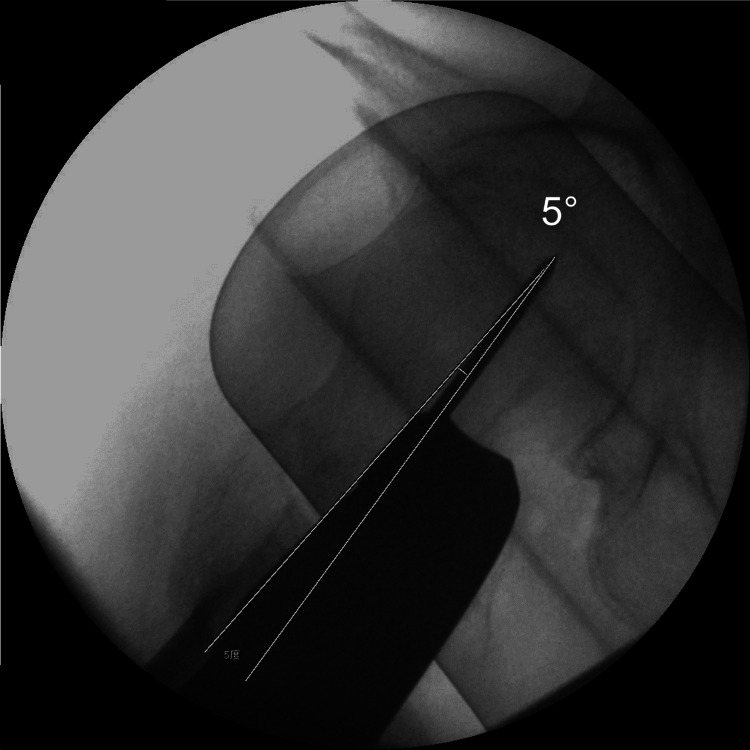
Axes of hip true lateral view Before inserting the lag screw, in the reference position, the true hip lateral view probably shows the difference between the neck axis and guide pin axis (example 5°).

During surgery, before inserting the lag screw, if the fractured femur is on the reference position, the true hip lateral view probably shows the difference between the neck axis and guide pin axis (Figure 9). This knowledge is helpful for calibrating each axis. In a follow-up study, we plan to use this knowledge for the evaluation of neck anteversion before inserting the lag screw during surgery. Furthermore, if we measure the neck anteversion of the normal femur on CT before surgery, the anteversion difference between the normal femur and fractured femur may be evaluated before inserting the lag screw, by measurement guide pin anteversion using an iPhone.

As a next step, we plan to evaluate the femoral anteversion using iPhone method before inserting the lag screw during surgery, and the bilateral differences of the femoral anteversion on CT after surgery.

Limitations

The reference position is not the true table-top-plane position because the flexion/extension direction level is determined by palpation and visual assessments. Furthermore, the table-top-plane is affected by the displacement of the greater trochanter. Osteoarthritis may change the original femoral posterior condyles axis of the individual. Visual confirmation of the reference image reappearance may cause a visual illusion. The support guide pin may bend while measuring with an iPhone. The only study outcome was the neck-shaft angle 125° nail’s outcome.

## Conclusions

Malrotation of the femur is defined as an internal/external rotation deformity > 15° relative to anteversion of the normal contralateral limb. The median difference between the AxP-LS-AV and 3DCT-LS-AV was 3.0°, and 40 (80%) patients had differences ≤ 5°. Use of an iPhone for intraoperative LS-AV measurement on table-top-plane standard had sufficient accuracy as an indicator of anteversion in femoral trochanteric fractures.
